# Metabotropic Glutamate Receptors at Ribbon Synapses in the Retina and Cochlea

**DOI:** 10.3390/cells11071097

**Published:** 2022-03-24

**Authors:** Lisa Klotz-Weigand, Ralf Enz

**Affiliations:** Institut für Biochemie, Friedrich-Alexander-Universität Erlangen-Nürnberg, 91054 Erlangen, Germany; lisa.janina.klotz@fau.de

**Keywords:** metabotropic glutamate receptor, retina, cochlea, photoreceptor, bipolar cell, inner hair cell, ribbon synapse, dimerization, protein interaction, short linear motif

## Abstract

Our senses define our view of the world. They allow us to adapt to environmental stimuli and are essential for communication and social behaviour. For most humans, seeing and hearing are central senses for their daily life. Our eyes and ears respond to an extraordinary broad range of stimuli covering about 12 log units of light intensity or acoustic power, respectively. The cellular basis is represented by sensory cells (photoreceptors in the retina and inner hair cells in the cochlea) that convert sensory inputs into electrical signals. Photoreceptors and inner hair cells have developed a specific pre-synaptic structure, termed synaptic ribbon, that is decorated with numerous vesicles filled with the excitatory neurotransmitter glutamate. At these ribbon synapses, glutamatergic signal transduction is guided by distinct sets of metabotropic glutamate receptors (mGluRs). MGluRs belong to group II and III of the receptor classification can inhibit neuronal activity, thus protecting neurons from overstimulation and subsequent degeneration. Consequently, dysfunction of mGluRs is associated with vision and hearing disorders. In this review, we introduce the principle characteristics of ribbon synapses and describe group II and III mGluRs in these fascinating structures in the retina and cochlea.

## 1. Ribbon Synapses

**Figure 1:**
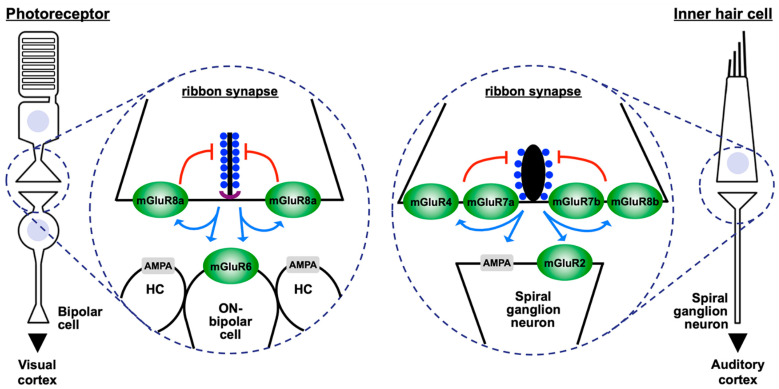
Inhibitory feed-back loops in sensory receptors of the retina and cochlea. Photoreceptors of the mammalian retina and inner hair cells of the cochlea form ribbon synapses that can be identified by the presence of an electron-dense pre-synaptic elongated structure close to the active zone. The circles (blue dashed lines) visualize enlargements of the ribbon synapses of both sensory cells. (**Left**) The pre-synaptic ribbon of photoreceptors is represented by a black bold vertical line that is anchored to the pre-synaptic membrane by proteins of the arciform density (purple). MGluR8a is the only glutamate receptor discovered to date at the photoreceptor pre-synapse. A subset of post-synaptic localized bipolar cells, termed “ON-bipolar cells” express mGluR6. Together with two post-synaptic processes of horizontal cells (HC), bipolar cell dendrites form a so-called triad at photoreceptor ribbon synapses. (**Right**) In contrast to the plate-like form of the pre-synaptic ribbon of photoreceptors, the ribbon of inner hair cells is more spherical. At inner hair cell ribbon synapses, mGluR4, mGluR7a, mGluR7b, and mGluR8b were found at the pre-synaptic site, while mGluR2 was localized at post-synaptic processes that are formed by spiral ganglion neurons. In both neuron types, synaptic vesicles filled with the excitatory neurotransmitter glutamate (blue spheres) are tethered to the sides of the ribbon. Upon stimulation of the sensory neurons, glutamate is released into the synaptic cleft, where it activates pre- and post-synaptic glutamate receptors (blue arrows). Activation of pre-synaptic mGluRs in photoreceptors and inner hair cells could initiate negative feed-back loops that inhibit the release of glutamate into the synaptic cleft (red lines). This, in turn, could reduce pre-synaptic activity which might prevent excitotoxic effects on post-synaptic neurons. In this sketch, only group II and III mGluRs are shown, while group I mGluRs present in bipolar cells and spiral ganglion neurons are not represented. As an example of post-synaptically localized ionotropic glutamate receptors, AMPA-type glutamate receptors are shown as grey rectangles, while ionotropic glutamate receptors of the NMDA and kainate type are not visualized for the sake of clarity. Piccolino, RIM, and other proteins associated with the ribbon are not shown.

Light and sound are two central sensory inputs that are based on the abilities of our eyes and ears to convert light signals or mechanical stimuli into neuronal signals. Despite the difference in the nature of these physical stimuli, the processing of visual and auditory inputs share similar mechanisms in the respective sensory tissues, the retina in the eye and the cochlea in the inner ear. These mechanisms involve a specialized chemical synapse that contains an electron-dense ribbon-like structure at its pre-synaptic site, which tethers numerous synaptic vesicles to its surface (Figure 1) [1,2]. In vertebrates, ribbon synapses are formed by sensory cells including photoreceptors and bipolar cells of the retina, hair cells of the cochlea and the vestibular system, and pinealocytes of the pineal gland [1]. Ribbons are immobilized at the pre-synaptic membrane from where they extend into the synaptic cytoplasm (Figure 1). Ribbon synapses seem to be perfectly suited to react to alterations in the strength of external stimuli with graded changes in a sustained release of glutamate. For ribbon synapses in the retina it has been suggested that graded changes of the membrane potential can encode a larger bandwidth of light intensities than would be possible by using action potentials [3].

Synaptic ribbon-like structures in the eye were described about 60 years ago [4,5]. The terminology “ribbon” originated from the bar-shaped appearance of these structures in cross-sections of the photoreceptor pre-synapse under the electron microscope. While ribbons differ in size and shape between cell types and species, their general anatomy and molecular composition is highly preserved [1,2]. Synaptic ribbons are mainly composed of the protein ribeye [6]. Ribeye is a splice variant of the C-terminal-binding protein 2 and thus belongs to the family of C-terminal-binding proteins [7,8]. Interestingly, ribeye has not been described at conventional synapses and thus seems to be specifically expressed at ribbon synapses [6]. Consequently, ribeye-deficient mice do not form ribbons at the pre-synaptic terminals of, e.g., photoreceptors or inner hair cells [9,10,11,12,13]. Interestingly, while the deletion of ribeye resulted in severe functional deficits in the retina, in the cochlea only minor hearing impairments were observed.

Ribbons are anchored to the pre-synaptic membrane of the active zone via a direct protein–protein interaction between ribeye and bassoon [14,15]. Bassoon is a major protein of the so-called “arciform density”, a structure present between the pre-synaptic membrane of the active zone and the ribbon that is visible in photoreceptors of the retina, but not in inner hair cells of the cochlea (Figure 1). Nevertheless, bassoon is also present at ribbon synapses of inner hair cells. Consequently, in mice lacking bassoon, non-anchored, so-called “free-floating”, ribbons were observed in the pre-synaptic terminals of photoreceptors and inner hair cells [16,17].

Piccolino is another protein that interacts with ribeye [18]. Piccolino results from alternative splicing of piccolo, which generates a premature stop codon causing a C-terminal truncation [19]. In retinae that lack expression of piccolino, the pre-synaptic ribbon structure changed to smaller aggregates of various forms, indicating a possible role of piccolino in the maintenance of the ribbon structure [20]. However, piccolino is most likely not the only molecule defining the shape of the ribbons. While photoreceptor ribbons have a plate-like appearance, inner hair cells of the cochlea contain more spherical-shaped ribbons, despite the expression of piccolino (Figure 1) [19,21,22,23,24]. A recent study used CRISPR-Cas9 to generate a complete knock-out of piccolo and piccolino. These mice showed severe anatomical and functional deficits in layers of the outer retina where the photoreceptors are located [25]. Interestingly, no changes were found in hair cells of the cochlea. Besides bassoon and piccolino, further proteins were suggested to be part of the ribbon complex, including Munc119, Tulp1, HIP3A, RIM and RIM-binding proteins, and liprin-alpha4 [2].

The exact function of pre-synaptic ribbons remains enigmatic. Given the localization of ribbons close to the active zone and the high number of tethered vesicles filled with glutamate, ribbons might organize and guide the arrangement of pre-synaptic vesicles for exocytosis [26]. Vesicles tethered to the ribbon are defined as the ribbon-bound pool of pre-synaptic vesicles, as opposed to the reserve pool of vesicles present in the pre-synaptic cytoplasm. If ribbon-bound vesicles are located at the base of the ribbon close to the pre-synaptic membrane they represent the readily releasable pool. In the retina it is well accepted that the ribbon-like structure increases the number of these readily releasable vesicles available for fusion with the pre-synaptic membrane, which ensures the high rates of tonic glutamate secretion observed in ribbon-containing photoreceptors and bipolar cells [8,27,28,29]. Indeed, a reduction of the readily releasable vesicle pool was shown in photoreceptors and bipolar cells of ribeye knockout mice [10,11]. In contrast, measuring changes in the membrane capacitance during exocytosis, inner hair cells of ribeye knockout mice did not show alterations in the readily releasable vesicle pool [9].

At the photoreceptor pre-synapse, thousands of synaptic vesicles filled with the neurotransmitter glutamate are tethered to the ribbons by thin filaments. Therefore, ribbons might function as a conveyer belt that facilitates transport and timing for the delivery of readily releasable synaptic vesicles to the pre-synaptic active zone. Other functions of pre-synaptic ribbons are discussed in the literature—ribbons might serve as scaffold structures that organize key proteins responsible for pre-synaptic glutamate release, including the clustering and localization of voltage-gated calcium channels at the active site [9,10,17,30]. An interesting idea is that the ribbon might allow synchronous fusion of several nearby vesicles, thus coordinating a multi-vesicular release of glutamate into the synaptic cleft, a process that was termed compound fusion [31,32,33,34].

## 2. Metabotropic Glutamate Receptors

Transmembrane receptors at the cell surface are key players in cellular communication. They function as cellular antennae that transmit information from extra- to intracellular compartments. The largest receptor family is formed by G-protein-coupled receptors (GPCRs) [35]. In humans, more than 800 GPCRs that are encoded by about 4% of our genome are known. In the central nervous system, GPCRs participate in most, if not all, physiological processes, including hearing and vision.

The family of GPCRs is organized in six groups that are designated A, B, C, frizzled, adhesion, and other [36]. Metabotropic glutamate receptors (mGluRs) belong to group C [37]. Group C receptors contain an extracellular N-terminal domain forming the ligand-binding pocket, seven transmembrane helices for membrane insertion, and an intracellular C-terminus of variable lengths that offers binding sites for regulatory proteins.

The mGluR family consists of eight members (mGluR1 to 8) that are organized in three groups (group I, II, and III), according to their sequence homology, sensitivity to drugs, and their coupling to intracellular signal pathways [38]. Each of the three groups shows clear preferences for pre- or post-synaptic sites. Group I contains mGluR1 and mGluR5 that are typically found at the post-synapse. MGluR2 and mGluR3 form group II and do not show any preference for a pre- or post-synaptic localization. Group III is the largest group and contains mGluR4, mGluR6, mGluR7, and mGluR8. Except for mGluR6, group III receptors are preferentially expressed at the pre-synaptic site. In general, group III receptors couple to intracellular pathways that are composed of heterotrimeric G-proteins, such as G_i_ or G_o_ that inhibit the activity of the adenylate cyclase and thereby reduce the intracellular concentration of the second messenger cAMP and the activity of the protein kinase A [39]. If these pathways are present in neurons, they need to be analyzed in every tissue individually. In cerebellar granule cells, e.g., it has been shown that the group III receptor mGluR7 couples also to the phospholipase-C pathway using the β/γ-subunits of the heterotrimeric G_o_ protein [40].

Most mGluRs are alternatively spliced in their intracellular C-terminus, which creates receptor isoforms, such as mGluR7a and mGluR7b. These isoforms contain multiple short linear sequence motifs in their isoform-specific C-termini, enabling a distinct regulation by interacting proteins [41,42]. By this means, mGluR C-termini and interacting proteins form signalling complexes that represent the molecular basis for a dynamic regulation of receptor function. A further diversity in receptor characteristics is achieved by the formation of homo- and heterodimeric receptors [43,44,45,46,47,48,49].

## 3. Metabotropic Glutamate Receptors at Ribbon Synapses in the Retina

In the mammalian retina, ribbon synapses are formed by photoreceptors and bipolar cells [50]. Rod photoreceptors are extremely sensitive to light and can respond to the energy of a single photon. Their pre-synapses can process changes of about five log units of luminosity [27]. Photoreceptor synapses contain plate-like ribbons that extend from the arciform density as a sheet of several hundred nanometres into the cytoplasm (Figure 1). The length of photoreceptor ribbons parallel to the pre-synaptic membrane can be up to one or two micrometres, providing docking places for thousands of synaptic vesicles [1,2].

The pre-synapse of rod and cone photoreceptors expresses mGluR8a that functions as a pre-synaptic auto-receptor for glutamate (Figure 1) [51,52]. Increasing concentrations of glutamate in the synaptic cleft activate the mGluR8a-associated signal cascade, which reduces pre-synaptic calcium concentrations and may thereby decrease neurotransmitter release [51,53]. Because of this inhibitory function at the first synapse in vision, mGluR8a was termed the “sunglass of the eye” [54]. In rods, the mGluR8a-associated signal cascade involves the β/γ-subunit of heterotrimeric G-proteins G_i2_ or G_t_ and Ca_v_1.4 L-type calcium channels [2,53]. The activity of mGluR8a-coupled heterotrimeric G-proteins might be regulated by additional intra- and/or extra-cellular protein interactions—intracellular proteins such as the cannabinoid receptor-interacting protein CRIP1 bind linear sequence motifs of mGluR8a and regulate, e.g., its endocytosis behaviour (see Section 6) [55]. Recently, the trans-synaptic proteins ELFN1 and ELFN2 were described to bind extracellular regions of mGluR8a, as well as of all other group III mGluR types [56,57]. For mGluR2, mGluR4, and mGluR6 (but not for mGluR8), a reduction of intracellular G-protein activity upon ELFN1 binding was reported [56]. An expression of the mGluR8b splice-variant has been analyzed in vertical cryostat sections of the mouse retina, but a presence of this receptor in photoreceptors was not observed [58].

The photoreceptor pre-synapse contacts three post-synaptic elements, formed by dendrites of bipolar and horizontal cells (Figure 1) [50]. A subtype of bipolar cells that is termed “ON-bipolar cell” expresses mGluR6 at its dendritic tips (Figure 1). This was first observed by Nomura and co-workers in 1994 [59]. MGluR6 is a central component of the scotopic pathway in retinal signal transduction, a pathway that integrates various proteins involved in night vision, including the G-protein Go, the regulators of G-proteins RGS7 and RGS11, the non-selective cation channel TRPM1, and the orphan G-protein-coupled receptor, GPR179 [60,61]. Consequently, mutations in mGluR6 and associated proteins cause congenital stationary night blindness [61,62]. Interestingly, mGluR6 is not found at ribbon synapses of the cochlea or at other chemical synapses in the brain, pointing to a specific requirement for this receptor in the visual system.

As photoreceptors, bipolar cells also contain ribbons at their pre-synapse. Ribbons of bipolar cells are much smaller than their counterparts in photoreceptors and can accommodate fewer synaptic vesicles [18]. In contrast to the ribbon-synapse in photoreceptors with its three post-synaptic elements, the pre-synapse of bipolar cells faces two post-synaptic structures [50]. These two post-synaptic structures are directly opposed to the two symmetrical halves of the pre-synaptic terminal, being located left and right of the ribbon in cross sections. Interestingly, mGluR7a was detected in the pre-synaptic membrane of bipolar cells at only one side of the ribbon, but absent from the other side [63]. This observation led to the idea that signals from the two pre-synaptic symmetrical halves are transmitted differently to the two post-synaptic neurons. Similar to the function of mGluR8a in photoreceptors, mGluR7a is suggested to function as auto-receptor in bipolar cells, regulating the secretion of glutamate into the synaptic cleft [64].

The remaining group III mGluR types, mGluR2 and mGluR4, were found exclusively post-synaptic to bipolar cell ribbon synapses, where they are expressed in just one of the two post-synaptic elements [65]. MGluR3 was not detected in the retina.

## 4. Metabotropic Glutamate Receptors at Ribbon Synapses in the Cochlea

The cochlea translates the mechanical fluctuations of sound waves into neuronal activity, covering a large range of frequency and intensity [66]. On the cellular level, this is ensured by a fast, temporal, and frequency-specific interplay between inner and outer hair cells. Outer hair cells enhance the pressure waves running along the cochlear axis by amplifying the movements of the basilar membrane, while inner hair cells transform these stimuli into neuronal activity.

Inner and outer hair cells of the cochlea form ribbon synapses that are essential for the fast and temporally specific signalling of these neurons. Ribbons of hair cells differ from their counterparts in the retina in many ways. In cross-section they are more spherical and thus are also named “dense bodies” (Figure 1). In general, they are smaller than ribbons in photoreceptors, their shape and size are variable even within the same hair cell, and they typically contain fewer vesicles [67,68].

While ribbon synapses in the retina contain two (bipolar cells) or three (photoreceptors) post-synaptic structures, each ribbon-containing pre-synapse of an inner hair cell contacts exactly one single post-synaptically localized dendrite of a spiral ganglion neuron [69]. This anatomical variability most likely mirrors the different physiological requirements of these neuron types. The retina processes visual information in vertically oriented parallel pathways [50]. At photoreceptor ribbon synapses, this processing starts with three post-synaptic elements formed by one bipolar and two horizontal cell dendrites (Figure 1). The neuronal activity of bipolar cells is then separated at bipolar cell ribbon synapses into two individual pathways formed by processes of amacrine and ganglion cells. Together, these parallel pathways assemble into neuronal networks that are essential for the generation of receptive fields and the selectivity in ganglion cell responses for various features of the visual information, including, e.g., object orientation and the direction of movement [70]. In the cochlea, one inner hair cell contains ribbon synapses with different anatomical and functional features, depending on their location on the modiolar or pillar side [71]. Compared to ribbon synapses on the modiolar side, ribbon synapses on the pillar side generally have smaller ribbons and respond to more hyperpolarized membrane potentials, which results in a lower threshold, a stronger calcium influx, and a larger release of glutamate. Recently it was shown that ribbon synapses on the pillar side show a tighter coupling between calcium influx and vesicular glutamate release than their counterparts on the modiolar side [72]. In line with this, AMPA receptor patches localized post-synaptically on spiral ganglion neurons are larger at the pillar side than at the modiolar side [73].

**Figure 2:**
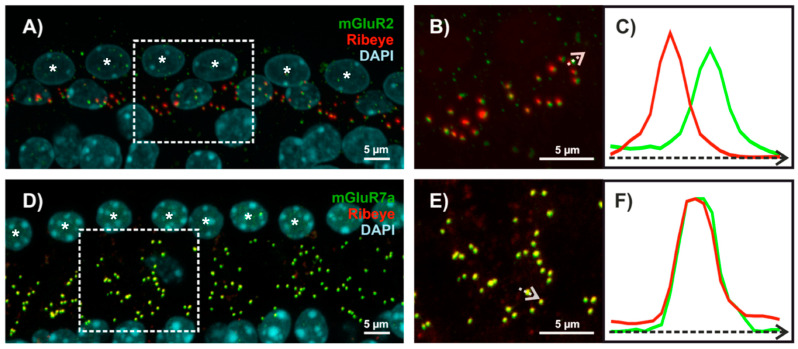
Localization of metabotropic glutamate receptors at ribbon synapses of inner hair cells. (**A**,**D**) Confocal z-projections of cochlear wholemounts of adult mice that were triple-labelled with specific antibodies for mGluR2 or mGluR7a (green), the pre-synaptic marker protein ribeye (red), and DAPI to visualize cellular nuclei (cyan). Fluorescent signals for mGluR2 are in close vicinity to pre-synaptic ribbons (**A**), while mGluR7a signals completely overlap with ribeye, resulting in yellow colour (**D**). Cellular nuclei of inner hair cells are labeled with asterisks. Panels (**B**,**E**) show enlarged views of fluorescent signals present in the two boxes indicated by white dashed lines in (**A**,**D**). Fluorescent signal intensities were measured along the white arrows and fluorescent profiles are compared in (**C**,**F**).

A typical inner hair cell forms about 10 to 15 ribbon pre-synapses, each contacting one spiral ganglion neuron [74]. Each ribbon synapse is sensitive to about six log units of signal intensity [27,66]. Analyzing cochlear wholemounts of gerbils and mice, several group II and III mGluRs were detected at this synapse type—mGluR4, mGluR7a, mGluR7b, and mGluR8b were present at the pre-synaptic site of inner hair cell ribbon synapses, while mGluR2 was detected at post-synaptic spiral ganglion neurons [75,76]. Stainings that demonstrate the different localization of mGluR2 and mGluR7a at ribbon synapses of inner hair cells are shown in Figure 2. MGluR3 and mGluR8a were not detectable at this synapse type. Thus, although photoreceptors and inner hair cells represent sensory neurons in the retina and cochlea that both form pre-synaptic ribbons, they do express completely different mGluR types at their pre-synaptic sites.

Not much data is available regarding intracellular signalling of mGluRs in inner hair cells of the cochlea. Two studies report expression of the Gαi subunit of heterotrimeric G-proteins in this cell type [77,78]. One study found that Gαi is involved in the elongation and shape of hair bundles [77]. Another study showed that inactivation of Gαi subunits by pertussis toxin increased the size of Ca_v_1.3 calcium-channel clusters and potentiated calcium influx at the inner hair cell pre-synapses [78]. This study also reports that the normally observed differences in calcium influx between the modiolar and pillar side of inner hair cells were lost. In addition, a genetic deletion of the Gαi3 subunit and of LGN/GPSM2, a modulator of G-protein signalling, abolished the differences in ribbon size between the modiolar and pillar sides. The authors suggest that signalling by Gαi and LGN/GPSM2 is an important factor to establish the modiolar/pillar gradient for principle properties of ribbon synapses of inner hair cells [78]. Given the reported expression of inhibitory Gαi proteins in inner hair cells and their function (as previously described) one can speculate that in principle the group III mGluRs detected at the inner hair cell pre-synapse (mGluR4, mGluR7a, mGluR7b, and mGluR8b) might couple to inhibitory Gαi proteins and thus could reduce the activity of the protein kinase A. Clearly, further studies are required to elucidate intracellular signalling mechanisms of mGluR types expressed at inner hair cells.

## 5. Pre-Synaptic Metabotropic Glutamate Receptors Are Central Elements of Inhibitory Feed-Back Loops at Ribbon Synapses

According to the concept of “glutamate excitotoxicity”, excessive glutamate concentrations in the synaptic cleft, caused, e.g., by noise trauma, might result in a degeneration and loss of synapses [79,80]. For example, spiral ganglion neurons post-synaptic to the inner hair cell ribbon synapse were protected from noise-induced elevated glutamate release upon block of AMPA receptor function [81]. In addition to post-synaptic mechanisms, inhibitory feedback loops present at pre-synaptic sites could reduce excessive glutamate secretion, thereby protecting neurons from cell death. The detected group III mGluR types in photoreceptors and inner hair cells are perfectly suited for this task, since they have been described to couple to intracellular pathways that reduce neuronal excitation. In doing so, these mGluR types could function as auto-receptors in photoreceptors and inner hair cells that limit pre-synaptic glutamate release (see red lines in Figure 1), thereby preventing excessive and toxic glutamate concentrations at ribbon synapses of these neurons.

The presence of mGluR7 isoforms at the inner hair cell ribbon synapse (Figure 2) [76] is highly interesting, because this receptor has been linked to age-related hearing-impairment and noise-induced hearing-loss [82,83]. Both receptors (mGluR7a and mGluR7b) show the lowest affinity for glutamate of all mGluR types, being in the millimolar range [38,43]. As a result, mGluR7 isoforms are believed to be activated only at high glutamate concentration in the synaptic cleft, which enables them to function as an intrinsic synaptic brake for excessive glutamate release [84].

**Figure 3:**
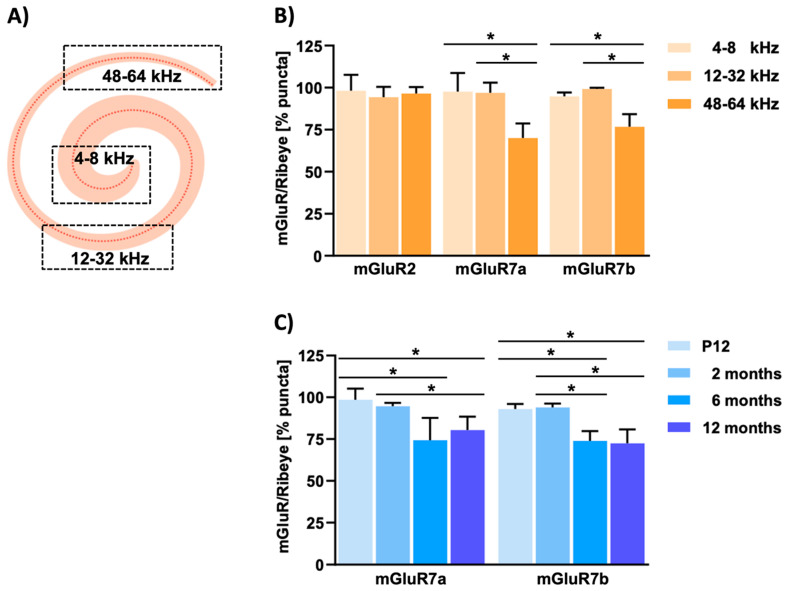
Expression of mGluR7 splice variants is reduced at ribbon synapses of inner hair cells in cochlear regions encoding high frequencies and with age. (**A**) Graphical representation of the cochlear anatomy. Red dots indicate hair cells located along the tonotopic axis of the cochlea. In the mouse, the apical region covers frequencies between 4 to 8 kHz, the middle region between 12 to 32 kHz, and the base is sensitive to frequencies up to 64 kHz. (**B**) The bar diagram shows the percentage of counted fluorescent puncta that represent the different frequencies visualized in different shades of orange normalized to the number of stained ribbons at inner hair cell synapses. The reduction of both mGluR7 isoforms at high frequencies is statistically significant. (**C**) Bar diagram showing the percentage of counted fluorescent puncta of mGluR7a and mGluR7b in mouse cochleae of different ages, as indicated by different shades of blue. Counted puncta were normalized to the number of stained ribbons at inner hair cell synapses, as in (**B**). In 6- and 12-month-old animals a significant reduction in the number of puncta representing mGluR7a and mGluR7b can be observed. Error bars are ± SD, statistical significances were calculated with the one-way ANOVA test, *p*-values < 0.05 are indicated by one asterisk (*).

To encode specific frequencies, inner hair cells form a single row along the cochlear axis in a tonotopic arrangement—inner hair cells located at the base are sensitive for high frequencies, while inner hair cells located at the apex encode lower frequencies (Figure 3) [69]. Interestingly, the number of ribbon synapses expressing mGluR7a or mGluR7b along the tonotopic axis of the mouse cochlea was significantly reduced in older animals and at higher frequencies (Figure 3) [76].

This observation could offer a molecular explanation for the described linkage of mGluR7 to age-related hearing-impairment and noise-induced hearing-loss [82,83,85,86,87]—after sound waves pass the oval window, they form a travelling wave along the tonotopic axis of the cochlea, with higher frequencies represented at the base and lower frequencies encoded towards the apex. This causes a persistent deflection of stereocilia in inner hair cells encoding higher frequencies at the base, which is not the case for inner hair cells located towards lower frequencies. As a consequence, inner hair cells encoding higher frequencies might be predisposed to stress factors, such as aging or noise trauma. It is therefore tempting to speculate that changes in the expression of mGluR7 are more risky for inner hair cells encoding higher frequencies, compared to inner hair cells encoding lower frequencies. Indeed, a single nucleotide polymorphism (SNP) that possibly increases mGluR7 expression was described to be protective for noise-induced hearing loss [83]. In this context, it is noteworthy that a recent study suggested the possibility that differences in behavioural studies of mGluR7 knockout mice might be at least partially caused by hearing deficits [88].

## 6. Protein Interactions of Metabotropic Glutamate Receptors at Ribbon Synapses

Originally, mGluRs were described as individually operating proteins. The elegant work of several laboratories proved this view to be an oversimplification. In fact, mGluRs represent central elements of large signalling complexes that integrate functionally related proteins, including other GPCR types, enzymes, and scaffold proteins [42,89].

Today, it is commonly accepted that mGluRs form constitutive dimers [43,44,45,46]. All mGluRs are able to form homodimers. In addition, members of group I, group II, and group III can heterodimerize [49]. For some mGluR combinations it was shown that heterodimer formation affects ligand affinity, receptor targeting, and G-protein signalling—for example, heterodimers composed of mGluR2 and mGluR3 showed a cooperative effect that increased their ligand sensitivity [45,90]. In mGluR2/mGluR4 heterodimers, G-protein signalling was mediated only by mGluR4 [47,91]. Furthermore, glutamate affinity was higher in mGluR2/mGluR7a heterodimers than in mGluR2 homodimers [43].

Given the distinct properties of heterodimeric versus homodimeric mGluRs, it is tempting to speculate that the diversity of mGluR functions at inner hair cell ribbon synapses could be further increased by heterodimerization of the receptors. MGluR4, mGluR7a, mGluR7b, and mGluR8b are localized at the pre-synaptic site of inner hair cell ribbon synapses [75,76] and in principle, heterodimer formation between these group III receptors is possible [44,49]. However, since heterodimers composed of combinations of mGluR4, mGluR7a, mGluR7b, and mGluR8b have, to date, not been characterized, distinct properties of these heterodimers remain speculative.

**Figure 4:**
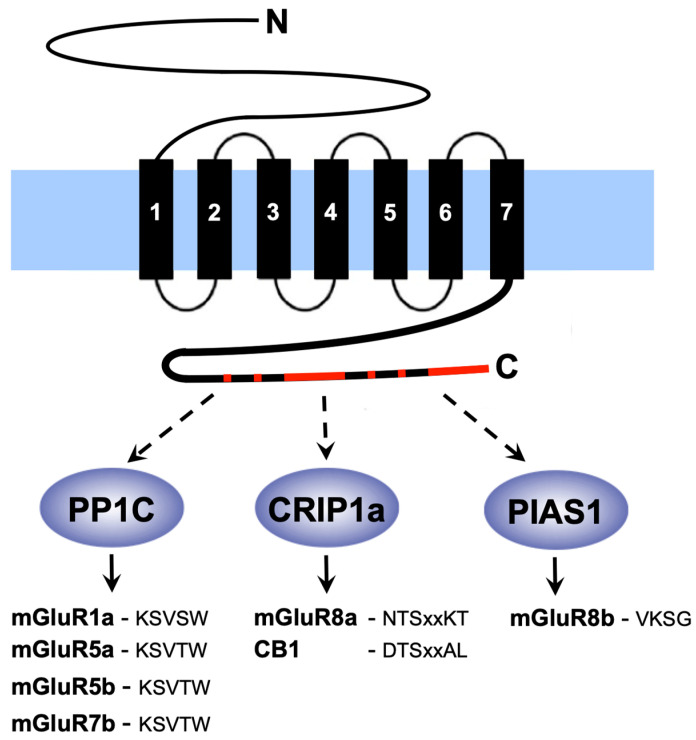
Intracellular C-termini of metabotropic glutamate receptors interact with cytosolic proteins in the retina. Sketch showing the membrane topology of one mGluR protein. The receptors are anchored in the cell membrane (blue rectangle) by seven transmembrane domains (black rectangles labeled 1 to 7). An extracellular N-terminal domain forms the ligand-binding pocket, while intracellular C-termini contain multiple short linear motifs (SLIMs—red) for interaction with cytosolic proteins (dashed arrows pointing to blue ovals). The catalytic subunit of protein phosphatase 1 (PP1C) binds the linear sequence KSVSW in the mGluR1a C-terminus (solid arrow), while mGluR5a splice-variants and mGluR7b C-termini contain the motif KSVTW. The SLIM in the mGluR8a C-terminus that contacts the cannabinoid receptor-interacting protein CRIP1a contains two amino acid positions of variable nature, indicated by “xx”. Presence of the SUMO E3 ligases PIAS1 enables SUMOylation of a lysine present in the motif VKSG in the mGluR8b C-terminus. Amino-acid sequences of SLIMs are given in the single letter code. CB1—cannabinoid receptor 1.

Numerous intracellular proteins that bind to the intracellular C-termini of mGluR types, thereby ensuring their precise regulation, both in space and in time, have been identified and characterized [92]. While mGluR-binding partners in inner hair cells of the cochlea have, to date, not been described, we identified several interactors in the retina that contact mGluR C-termini in an isoform-specific manner (Figure 4)—a catalytic subunit of protein phosphatase 1 (PP1C) contacts the C-termini of mGluR1a, mGluR5a, mGluR5b, and mGluR7b, but does not interact with mGluR1b and mGluR7a [93,94,95,96]. The SUMO E3-ligases PIAS1 showed a high affinity for mGluR7a, mGluR7b, and mGluR8b, but not for mGluR8a, and SUMOylation of mGluR8b was demonstrated in a cellular context [97]. Besides PIAS1, this study also identified other E3 ligases (PIAS3L, Pc2) as interaction partners of mGluRs. Furthermore, a protein known to interact with and regulate the cannabinoid receptor 1 (cannabinoid-receptor-interacting protein 1a-CRIP1a) binds specifically to the mGluR8a C-terminus, but not to mGluR8b [55].A well-established concept in the life sciences is that structure determines function. While this holds also true for proteins, many proteins contain unstructured regions [98]. These intrinsically disordered regions are predestined to use so-called short linear motifs (SLIMs) for protein–protein interactions [99]. Crystal structures of intracellular domains of glutamate receptors were not described [100,101,102,103,104], indicating that these domains are intrinsically disordered and highly flexible receptor regions. Indeed, a combination of biochemical and biophysical techniques showed that the intracellular C-termini of mGluR6, mGluR7a, and mGluR8a do not form secondary or tertiary structures that remain stable over time [105]. Rather, it is assumed that these mGluR C-termini adopt defined 3D structures only upon binding to regulatory proteins. This feature enables these receptors to harbour overlapping binding motifs for distinct interactors.

The 3D structure of some SLIMs present in mGluR C-termini was reported in contact with their binding partners [89]. A SLIM composed of five amino acids present in the splice-specific region of the mGluR7b C-terminus (Lys-Ser-Val-Thr-Trp) (Figure 4) contacts PP1C in an extended conformation [94,95]. The five amino acids of this motif show distinct binding characteristics–the two hydrophobic amino acids (Val and Trp) dip into hydrophobic pockets present on the surface of the protein phosphatase 1. This positions the mGluR7b C-terminus in the correct way to allow polar interactions between the three polar residues of the motif (Lys, Ser, and Thr) and corresponding amino acids of the enzyme.

A SLIM of seven amino acids present in the C-terminal domain of the cannabinoid receptor 1 contacts CRIP1a (Figure 4) [55]. A conserved amino acid motif is present in the C-terminus of mGluR8a, but not of mGluR8b. Indeed, endogenous endocytosis of the cannabinoid receptor 1 and of mGluR8a, but not of mGluR8b, was reduced in the presence of CRIP1a, pointing to a regulation in receptor turnover by CRIP1a [55]. This finding offers the exciting possibility that CRIP1a might represent a regulatory protein that could act synergistically on the cannabinoid receptor 1 and on mGluR8a. Interestingly, photoreceptors express both GPCR types at their pre-synapse [51,106,107]. Therefore, it would be interesting to test if mGluR8a and the cannabinoid receptor 1 form heterodimeric receptors at ribbon synapses of photoreceptors.

## 7. Conclusions

Ribbon synapses are fascinating nanomachines formed by sensory cells. These synapses respond to changes in sensory input with graded alterations in their tonic glutamate release. Ribbon synapses in the retina and cochlea contain many mGluR types at their pre- and post-synaptic sites. These receptors guide the glutamate secretion into the synaptic cleft, regulate the activity of post-synaptic neurons, and can protect pre- and post-synaptic neurons from glutamate excitotoxicity. In order to understand functional properties of these receptors, detailed knowledge of their localization, their dimerization status, and their regulation by interacting proteins is essential.

While in photoreceptors, just one pre-synaptic glutamate receptor (mGluR8a) was observed, inner hair cells of the cochlea express mGluR4, mGluR7a, mGluR7b, and mGluR8b at their pre-synapse (Figure 1). It will be interesting to analyze if these receptors occupy distinct regions in respect to the synaptic ribbon of inner hair cell pre-synapses. Ribbon synapses at different locations of the inner hair cell body (modiolar or pillar side) show differences in threshold, influx of calcium, and glutamate release, as outlined in Section 4. The distribution of AMPA receptors localized post-synaptically on spiral ganglion neurons also varies, depending on the ribbon synapse contacted. As a result, different spiral ganglion neurons show different susceptibilities for noise trauma [108]. Thus, it will be of interest to elucidate if the localization of mGluR4, mGluR7a, mGluR7b, and mGluR8b changes between ribbon synapses located at the modiolar or pillar side of inner hair cells.

Moreover, it would be fascinating to analyze if pre-synaptically localized mGluR4, mGluR7a, mGluR7b, and mGluR8b might form heterodimers at inner hair cell ribbon synapses. Since heterodimers might have functions distinct from their homodimeric counterparts, as outlined in Section 6, heterodimeric receptors could represent new drug targets for the design of new clinical strategies that protect inner hair cells from overstimulation, glutamate excitotoxicity, and hearing disorders. In addition, obtained data describing the exact synaptic localization of these receptors and their dimerization properties paves the ground for designing experiments to elucidate the function of these mGluRs in the cochlea.

A further important element is the regulation of homo- and/or heterodimeric mGluRs by interacting proteins. While some interactors for group III mGluRs were described in the retina, it is not known which proteins interact and regulate mGluR4, mGluR7a, mGluR7b, and mGluR8b in inner hair cells. Once identified, contact regions between the receptors and their binding proteins need to be mapped and their 3-dimensional geometry needs to be solved. This remains a challenging task, since mGluR C-termini seem to be intrinsically disordered and adopt to a specific geometry only after binding to interacting proteins. This high structural flexibility in the isolated intracellular mGluR C-termini seems to be a requirement for the proper biological function of these receptors. Thus, analyzing the geometry of the mGluRs in contact with regulatory proteins opens the possibility of observing these signalling complexes “at work”. Based on this structural information, small molecules can be designed that might either inhibit, or stabilize protein interactions of the receptors. Ultimately, these studies might be helpful in developing new therapeutic strategies for the treatment of neurodegenerative diseases in the eye and the ear.

## Data Availability

Not applicable.
